# Loss of One or Two *PATZ1* Alleles Has a Critical Role in the Progression of Thyroid Carcinomas Induced by the *RET/PTC1* Oncogene

**DOI:** 10.3390/cancers10040092

**Published:** 2018-03-27

**Authors:** Mario Monaco, Giuseppe Palma, Michela Vitiello, Anna Capiluongo, Barbara D’Andrea, Emilia Vuttariello, Antonio Luciano, Laura Cerchia, Gennaro Chiappetta, Claudio Arra, Alfredo Fusco, Monica Fedele

**Affiliations:** 1 Dipartimento di Ricerca Traslazionale a Supporto dei Percorsi Oncologici, S.C. Genomica Funzionale, Istituto Nazionale Tumori—IRCCS—Fondazione G. Pascale, 80131 Naples, Italy; m.monaco@istitutotumori.na.it (M.M.); capiluongoanna@gmail.com (A.C.); e.vuttariello@istitutotumori.na.it (E.V.); g.chiappetta@istitutotumori.na.it (G.C.); 2 S.S.D. Sperimentazione Animale, Istituto Nazionale Tumori—IRCCS—Fondazione G. Pascale, 80131 Naples, Italy; giuseppe.palma@istitutotumori.na.it (G.P.); a.luciano@istitutotumori.na.it (A.L.); c.arra@istitutotumori.na.it (C.A.); 3 CNR—Institute of Experimental Endocrinology and Oncology (IEOS), 80131 Naples, Italy; michela.vitiello@gmail.com (M.V.); cerchia@unina.it (L.C.); 4 Centro Medico Polispecialistico (CMO), 80100 Naples, Italy; barbara.dandrea@gmail.com; 5 Department of Molecular Medicine and Medical Biotechnologies, University of Naples “Federico II”, 80131 Naples, Italy; alfusco@unina.it

**Keywords:** thyroid cancer, *PATZ1*, *RET/PTC*, mice, solid variant, anaplastic

## Abstract

POZ/BTB and AT-hook-containing zinc finger protein 1 (PATZ1) is an emerging cancer-related gene that is downregulated in different human malignancies, including thyroid cancer, where its levels gradually decrease going from papillary thyroid carcinomas (PTC) to poorly differentiated and undifferentiated highly aggressive anaplastic carcinomas (ATC). The restoration of PATZ1 expression in thyroid cancer cells reverted their malignant phenotype by inducing mesenchymal-to-epithelial transition, thus validating a tumor suppressor role for PATZ1 and suggesting its involvement in thyroid cancer progression. Here, we investigated the consequences of the homozygous and heterozygous loss of PATZ1 in the context of a mouse modeling of PTC, represented by mice carrying the *RET/PTC1* oncogene under the thyroid specific control of the thyroglobulin promoter RET/PTC1 (RET/PTC1^TG^). The phenotypic analysis of RET/PTC1^TG^ mice intercrossed with Patz1-knockout mice revealed that deficiency of both *Patz1* alleles enhanced thyroid cancer incidence in RET/PTC1^TG^ mice, but not the heterozygous knockout of the *Patz1* gene. However, both RET/PTC1^TG^;Patz1^+/−^ and RET/PTC1^TG^;Patz1^−/−^ mice developed a more aggressive thyroid cancer phenotype—characterized by higher Ki-67 expression, presence of ATCs, and increased incidence of solid variants of PTC—than that shown by RET/PTC1^TG^; Patz1^+/+^ compound mice. These results confirm that PATZ1 downregulation has a critical role in thyroid carcinogenesis, showing that it cooperates with RET/PTC1 in thyroid cancer progression.

## 1. Introduction

Thyroid cancer is the most common type of endocrine malignancy, and one of the few tumor types for which incidence has been increasing over the past 20 years and is predicted to be the fourth leading cancer diagnosis by 2030 [1]. Thyroid carcinomas derived from follicular epithelial cells include well-differentiated carcinomas, represented by papillary thyroid carcinomas (PTC) and follicular thyroid carcinomas (FTC); poorly differentiated thyroid carcinomas (PDTC); and anaplastic thyroid carcinomas (ATC). Incidence rates of thyroid cancer widely vary worldwide. PTC is the predominant form of thyroid cancer in both adults and children (ranging from 65% in Ireland to 93% in Japan and Korea), and together with the less frequent FTC (6–10% of the cases) has a favorable prognosis with a five-year prognosis higher than 90% [2]. Conversely, PDTC and ATC, constituting only about 1–2% of all thyroid cancers, are highly aggressive and account for the majority of deaths from thyroid carcinoma [3]. 

As far as the PTC development is concerned, most of the initiating events have been determined. Indeed, RET/PTC rearrangements are present in about 30% of the cases: they consist in the fusion of the RET proto-oncogene tyrosine kinase (TK) with other genes that provide 5’ coding region and the promoter, thus allowing the expression of the RET TK in thyroid follicular cells. Even though several RET/PTC isoforms have been isolated, only the RET/PTC1 and RET/PTC3 isoforms are present in a significant number of PTC cases [4]. RET/PTC1 is generated by the fusion of RET TK domain with the 5’ terminal region of the *CCDC6/H4* gene, and is associated with the classical PTC variant [5], whereas RET/PTC3 consists in the fusion between the TK domain of RET and the *RFG/NCOA4* gene [6], and is usually associated with a more aggressive phenotype, particularly with the solid variant; bigger size; and a more rapid development of the tumor [7]. Other common genetic alterations in PTCs, mutually exclusive with RET rearrangements, are BRAF point mutations (about 40% of the cases) and TRK rearrangements (5%). Ras mutations, including all the members of this family, are present in about 5% of PTC [8]. 

The POZ/BTB and AT-hook-containing zinc finger protein 1 (PATZ1) is a member of the POZ and Kruppel-like (POK) family of architectural transcription factors and is involved in several physiological and pathological processes, including development and cancer [9]. Recent results evidence a role for PATZ1 as tumor suppressor in thyroid cancer, involved in both carcinogenesis and cancer progression of thyroid follicular cells [10,11]. Indeed, its expression is abundant in the nucleus of normal follicular thyroid cells, whereas it is increasingly downregulated and/or delocalized in the cytosol of neoplastic thyroid cells going from PTC toward PDTC and finally to ATC [10,11]. Moreover, the restoration of its expression in human carcinoma cell lines reverted the malignant phenotype likely by regulating the expression of several genes—including *EpCam*, *RhoE*, *Caldesmon*, *MMP9*, *MMP2*, and *uPa*—involved in epithelial–mesenchymal transition (EMT), cell migration, and invasion [10,11]. However, in vivo evidence of a role of PATZ1 in thyroid tumor progression is limited to ATC mouse xenografts, in which PATZ1 expression was associated with a partial mesenchymal–epithelial transition (MET) [10]. Conversely, there is lack of information about the role of PATZ1 in animal models of PTC. 

Transgenic mice overexpressing the *RET/PTC1* oncogene under the transcriptional control of thyroglobulin promoter have been generated [12,13]. These mice develop bilateral thyroid carcinoma with cellular features of human PTC, with the presence of neoplastic nodules with a macropapillary architecture within a macrofollicular area. Noteworthily, they are not invasive in the surrounding tissues nor is evidence of distant or lymph-node metastasis observed [13]. Also, at the cytological level, they show typical features of the papillary subtype of thyroid carcinoma, including nuclear polymorphism with nuclear grooves, ground glass nuclei, and mitoses [12,13]. Conversely, the phenotype of the RET/PTC3 transgenic mice is characterized by the development of the solid variant, but also in this case no distant metastases were observed [14]. Only the additional deletion of p53 causes invasiveness with the presence of distant metastases in RET/PTC1 mice [15], or a poorly differentiated and high proliferative phenotype in RET/PTC3 mice [16], confirming the key role of p53 in PTC progression toward a more aggressive and less differentiated phenotype [17]. Similarly, a solid variant and a less differentiated phenotype were observed in RET/PTC1 knockout for the *cl2*/*ccdc80* gene [18], while ATCs were developed by thyroid-specific inactivation of p53 and Pten [19], or thyroid-specific combined mutations of BRAF^V600E^ and PIK3CA^H1047R^ [20], confirming the multi-step carcinogenesis model.

In this study, we generated mice characterized by thyrocyte-specific expression of the *RET/PTC1* oncogene in conjunction with one or two null alleles of *Patz1*. The absence or even the reduction of Patz1 expression enhanced RET/PTC1-induced thyroid carcinogenesis, promoting the rapid development of aggressive carcinomas, including ATC and solid variant of PTC. Therefore, this genetically engineered mouse model recapitulates key features of the human ATC and aggressive PTC and may represent a suitable model for the development of innovative therapeutic approaches.

## 2. Results

### 2.1. RET/PTC1 Transgenic Mice Show Higher Incidence of Thyroid Carcinomas in Absence of PATZ1

RET/PTC1-transgenic (RET/PTC1^TG^) mice develop slowly progressive thyroid tumors displaying the cytohistological aspects of human PTC [12,13]. Patz1-knockout mice, either heterozygous or homozygous for a Patz1-null mutation [21], have an increased susceptibility to develop malignant tumors, including lymphomas, sarcomas, hepatocellular carcinomas, and lung adenocarcinomas [9].

**Figure 1:**
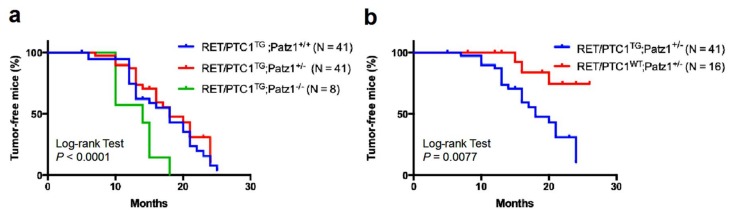
Tumor incidence curves. (**a**) Kaplan–Meier analysis of the effect of progressive Patz1 deletion on the thyroid tumor incidence of RET/PTC1 mice. The comparison of the survival curves by log-rank test showed they were significantly different (*p* < 0.0001), with the worst outcome in RET/PTC1 mice carrying the homozygous deletion of the *Patz1* gene. Comparison of single curves by log-rank (Mantel–Cox) test revealed significant differences between RET/PTC1^TG^;Patz1^−/−^ and either RET/PTC1^TG^;Patz1^+/+^ or RET/PTC1^TG^;Patz1^+/−^ mice (*p* = 0.029 and 0.0032, respectively), whereas no significant differences resulted between RET/PTC1^TG^;Patz1^+/−^ and RET/PTC1^TG^;Patz1^+/+^ mice (*p* = 0.36); (**b**) Kaplan–Meier analysis of Patz1^+/−^ mice with or without *RET/PTC1* transgene were compared by log-rank test: they were significantly different (*p* = 0.0077).

Building on our previous results, showing that PATZ1 is downregulated in human thyroid carcinomas and plays a tumor suppressor role in thyroid cancer cells by inhibiting their malignant phenotype [10,22], we intercrossed RET/PTC1^TG^ [13] with Patz1-knockout mice [21] to better define the role of PATZ1 in thyroid carcinogenesis driven by the *RET/PTC1* oncogene. As shown in Figure 1a, double mutant RET/PTC1^TG^;Patz1^−/−^ mice developed thyroid tumors with higher incidence (100%) and earlier onset (median age of tumor incidence of 14 months) than their RET/PTC1^TG^;Patz1^+/+^ compound mice (54% of tumors at a median age of 18 months) (*p* = 0.0105, Log-rank (Mantell-Cox) test; HR = 0.3831; 95% CI 0.05877–0.6138). Conversely, no significant differences were observed between the survival curves of RET/PTC1^TG^;Patz1^+/−^ and RET/PTC1;Patz1^+/+^ mice (Figure 1a), even though, in the new genetic background resulting from the intercrossing of the two mutants, loss of a single Patz1 allele caused the development of PTC in 3 out of 16 mice, but with a better survival curve than that observed in RET/PTC1^TG^;Patz1^+/−^ mice (*p* = 0.0077, Log-rank (Mantell-Cox) test; HR = 4.210; 95% CI 1.412–7.199) (Figure 1b).

### 2.2. Loss of One or Two Patz1 Alleles Enhances Thyroid Tumor Aggressiveness in RET/PTC1 Mice

In the time-window of 10–17 months of age, thyroid carcinomas were present in 100% of RET/PTC1^TG^;Patz1^−/−^, whereas they were diagnosed in 54% and 58% of RET/PTC1^TG^;Patz1^+/+^ and RET/PTC1;Patz1^+/−^ mice, respectively, that also show the presence of hyperplasia/goiter (Table 1). 

**Figure 2:**
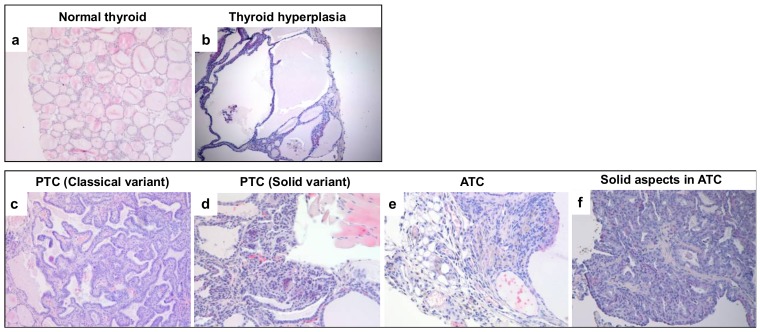
Histopathological features of tumors developed by RET/PTC1^TG^;Patz1-ko mice. Representative images of (**a**) normal thyroid as it appeared in a wild-type mouse. Uniform colloid-filled follicles, composed of a one cell thick layer of cuboidal epithelium are present; (**b**) hyperplastic thyroid lesion as it appeared in a RET/PTC1^TG^;Patz1^+/+^ mouse. Increased numbers of thyroid follicular epithelial cells surrounding large colloid-deficient follicles are present; (**c**) a PTC (classical variant) developed in a RET/PTC1^TG^;Patz1^+/−^ mouse. Follicular epithelial cells cluster together forming continuous sheets or papillae that surround a fibrovascular stalk; (**d**) a PTC (solid variant) as it appeared in a RET/PTC1^TG^;Patz1^−/−^ mouse. Note the presence of large regions of tissue devoid of follicles or papillae, characteristic of this variant; (**e**) ATC found in a RET/PTC1^TG^;Patz1^+/−^ mouse. The cells appear irregularly arranged in a mass with solid aspects; (**f**) solid aspects in the same ATC shown in (**e**). Original magnification: ×10 in (**a**–**c**); ×20 in (**d**–**f**).

The thyroid carcinomas developed by these mice closely phenocopy the human pathology. As shown in Figure 2, uniform colloid-filled follicles, composed of a one cell thick layer of cuboidal epithelium are present in normal thyroids (Figure 2a), while increased numbers of thyroid follicular epithelial cells surrounding large colloid-deficient follicles are present in hyperplastic lesions (Figure 2b). Follicular epithelial cells that cluster together forming continuous sheets or papillae were observed in classical variants of PTCs (Figure 2c), while large regions of tissue devoid of follicles or papillae were present in the solid ones (Figure 2d). Interestingly, a solid variant of PTC found in RET/PTC1^TG^;Patz1^+/−^ mice developed lymph-node metastases.

Notably, 3 out of 14 tumors (21%) in RET/PTC1^TG^;Patz1^+/−^ mice showed features of either ATC or PDTC, whereas only one PDTC—but no ATC—was found in RET/PTC1^TG^;Patz1^+/+^. The pathological features of the PDTCs were those of the solid type, but without the nuclear features of PTC, and with the presence of necrosis and high mitotic activity (data not shown). Spindle cell morphology, with frequent giant cells, was observed in ATCs (Figure 2e). Interestingly, in both cases of ATC developed by RET/PTC1^TG^;Patz1^+/−^ mice, the anaplastic phenotype co-existed with a solid variant of PTC (Figure 2f), suggesting that it has arisen from pre-existing aggressive papillary carcinomas. 

**Figure 3:**
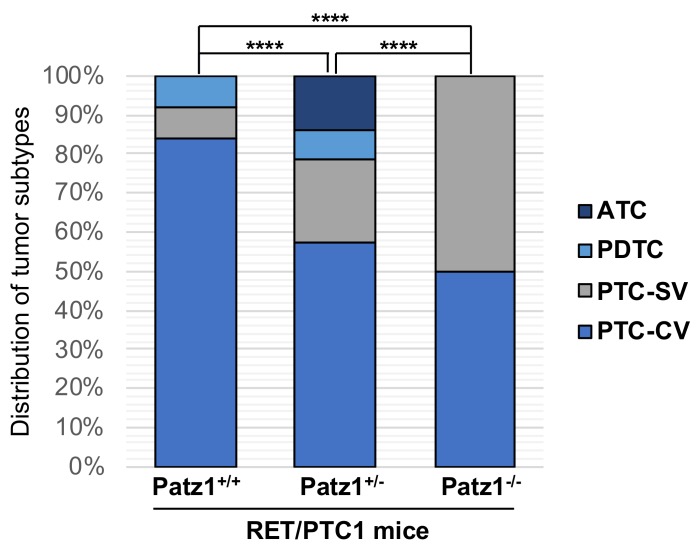
Tumor subtype spectrum in RET/PTC1^TG^ mice with or without heterozygous or homozygous deletion of the *Patz1* gene. **** *p* < 0.0001 as assessed by Pearson’s *x*^2^ test. PTC-SV, solid variant of PTC; PTC-CV, classical variant of PTC.

In summary, the spectrum of thyroid malignant neoplasms developed by RET/PTC1^TG^ mice carrying one, two, or no *Patz1-null* alleles included classical and solid variants of PTC, PDTCs, and ATCs (Figure 3). Importantly, at the observed time-window of age, the more aggressive solid variant of PTC was present in 3 out of 14 carcinomas (21%) in RET/PTC1;Patz1^+/−^ mice, compared with 1 out of 12 carcinomas (8%) in RET/PTC1^TG^;Patz1^+/+^ mice. Both classical and solid variants of PTC were present in equal percentage in RET/PTC1^TG^;Patz1^−/−^, while the classical variant was predominant on the solid one in either RET/PTC1^TG^;Patz1^+/−^ (~3:1 ratio) or RET/PTC1^TG^;Patz1^+/+^ (10:1 ratio) (Figure 3). Therefore, even though heterozygous loss of Patz1 does not change the time-dependent incidence of thyroid tumors in RET/PTC1^TG^ mice, histopathological analyses revealed a significant difference in thyroid tumor phenotype distribution developed in RET/PTC1^TG^;Patz1^+/−^ mice compared to compound RET/PTC1^TG^;Patz1^+/+^ mice (*p* < 0.0001, chi-square test) and RET/PTC1^TG^;Patz1^−/−^ (*p* < 0.0001, chi-square test) (Figure 3), indicating a progressive cooperation between Patz1 allelic loss and RET activation toward an increasingly malignant phenotype resulting in the development of aggressive, metastatic tumors closely resembling human thyroid carcinomas.

### 2.3. Patz1-Null Mutation Enhances Proliferation of Thyroid Cancer Cells in RET/PTC1^TG^ Mice 

**Figure 4:**
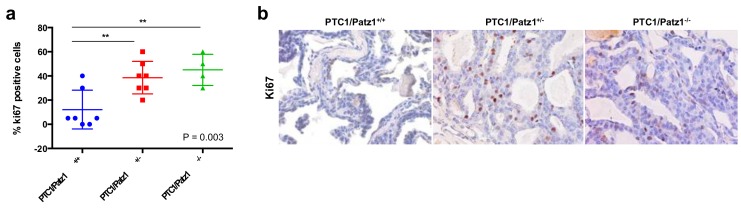
(**a**) Percentage of tumor cells stained positively for Ki-67 proliferation marker in RET/PTC1^TG^ mice with different Patz1 genotype. Differences among means were statistically significant, according to one-way ANOVA analysis of variance (*p* value is indicated on the bottom-right corner of the graph). ** *p* < 0.01, according to Tukey’s multiple comparisons test. *n* = 7 for either RET/PTC1^TG^;Patz1^+/+^ or RET/PTC1^TG^;Patz1^+/−^ and 4 for RET/PTC1^TG^;Patz1^−/−^; (**b**) Representative Ki-67 staining of thyroid carcinomas from RET/PTC1^TG^;Patz1^+/+^, RET/PTC1^TG^;Patz1^+/−^, and RET/PTC1^TG^;Patz1^−/−^ mice. Original magnification: ×40.

To deeper investigate phenotypic differences in RET/PTC1^TG^ mice carrying one, two, or no *Patz1-null* alleles, we analyzed expression of Ki-67 (a typical marker of cell proliferation) by immunohistochemistry on tumor samples from each genotypic group. The results have shown a significant increase in the percentage of Ki-67 positive cells (*p* < 0.01; Tukey’s multiple comparisons test) in thyroid tumors from either RET/PTC1^TG^;Patz1^+/−^ or RET/PTC1^TG^;Patz1^−/−^ mice, compared to those from RET/PTC1^TG^;Patz1^+/+^ mice. Conversely, no significant differences were observed between RET/PTC1^TG^;Patz1^−/−^ and RET/PTC1^TG^;Patz1^+/−^ mice (Figure 4). These results indicate a cooperative role of Patz1-null mutation with RET/PTC1 in enhancing thyroid cancer cell proliferation, accounting for the higher aggressive phenotype of tumors developed by RET/PTC1^TG^ mice, heterozygous or homozygous for the Patz1-knockout mutation, compared to single mutant RET/PTC1^TG^ mice. 

**Figure 5:**
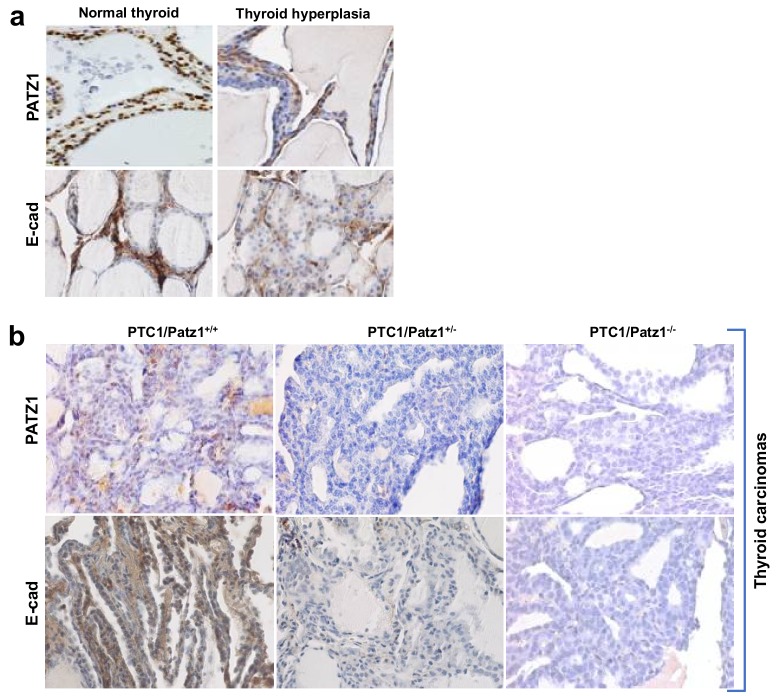
Representative mouse thyroid samples immunostained for PATZ1 and E-cadherin. (**a**) normal and hyperplastic thyroids show positive immunostaining for both PATZ1 and E-cadherin; (**b**) thyroid carcinomas from RET/PTC1^TG^;Patz1^+/+^, RET/PTC1^TG^;Patz1^+/−^, and RET/PTC1^TG^;Patz1^−/−^ mice stained for PATZ1 and E-cadherin. Original magnification: ×40.

Interestingly, PATZ1 expression was either reduced or lost in thyroid carcinomas derived by either RET/PTC1^TG^;Patz1^+/+^ or RET/PTC1^TG^;Patz1^+/−^ mice, respectively, compared to normal thyroids or hyperplastic lesions (Figure 5). In particular, PATZ1 positivity was associated with more differentiated tumor areas in a same papillary tumor with solid aspects (data not shown). This is consistent with previous results in human thyroid carcinomas [11,12], and may explain why we did not observe significant differences in the proliferation index of RET/PTC1^TG^;Patz1^+/−^ and RET/PTC1^TG^;Patz1^−/−^ thyroid cancers. According to our previous data showing that the maintenance of PATZ1 expression in thyroid cancer cells was associated with expression of E-cadherin [11], we found that the rare cases of PATZ1 retention in thyroid cancer cells were associated with immuno-histochemical detection of E-cadherin, which was not or barely detectable in all the other thyroid cancer samples analyzed, where also PATZ1 expression was lost (Figure 5).

## 3. Discussion

It has been previously reported that PATZ1 is downregulated in thyroid cancer cell lines and tissues compared to normal thyroid cell lines and tissue, and its expression is inversely correlated with the degree of malignancy of thyroid carcinomas being lower in PDTC and ATC compared with PTC [10,11,23], then suggesting a tumor suppressor role of PATZ1 in the progression from PTC to ATC. Such a hypothesis has been supported by in vitro studies showing that restoration of PATZ1 in rat and human malignant thyroid cells, including PTC and ATC cell lines, inhibits cell proliferation, migration, and invasion, that are, conversely [10,11,23], enhanced by PATZ1 silencing in both normal and malignant thyroid cells [11]. 

Then, in order to validate the tumor suppressor role of PATZ1 in thyroid carcinogenesis in vivo, we crossed a mouse model of PTC, carrying the *RET/PTC1* oncogene under the thyroid-specific control of the bovine thyroglobulin promoter [13] with mice knockout for the *Patz1* gene [21]. 

In humans, PTC can be subdivided in several histologic variants, showing distinct patterns of growth and clinical behavior, which include: (i) classical, with papillary architecture and Psammona bodies (scarred and calcified remnants of infarcted papillae), the most common; (ii) follicular, with cells organized in follicles, accounting for approximately 10% of all PTCs; (iii) oncocytic or Hurthle-cell, characterized by cells with abundant eosinophilic granular cytoplasm as a result of accumulation of altered mitochondria, accounting for about 3–10% of all differentiated thyroid cancers, and present also as a variant of FTC; (iv) tall-cell, with cells two to three times as tall as they are wide, showing abundant eosinophilic cytoplasm, occurring in about 10% of PTCs; (v) cribriform morular, associated with familial adenomatous polyposis, with interspersed balls of squamoid cells or morules; (vi) solid, characterized by solid sheets, more common in children and associated with the Chernobyl nuclear accident and sometimes defined as poorly differentiated carcinoma with insular patterns; (vii) columnar, with elongated nuclei in tall cells, very rare [24]. 

Our results show that homozygous deletion of the *Patz1* gene worsens outcome in RET/PTC1 mice, since RET/PTC1^TG^;Patz1^−/−^ mice develop thyroid carcinomas four months earlier than RET/PTC1^TG^;Patz1^+/+^ controls, and induces a thyroid cancer phenotype characterized by the presence of a higher number of proliferating cells and an increased incidence of the solid variant with respect to controls. Interestingly, RET/PTC1^TG^ mice heterozygous for the Patz1-knockout mutation do not significantly differ from RET/PTC1^TG^;Patz1^+/+^ control mice as far as thyroid tumor incidence is concerned, but their thyroid cancer phenotype is significantly more aggressive than that of controls. Indeed, it is undistinguishable from that of RET/PTC1^TG^;Patz1^−/−^ in terms of Ki-67 expression. Moreover, these mice have developed ATCs and an increased number of solid variants of PTC compared to RET/PTC1^TG^;Patz1^+/+^ compounds. Therefore, RET/PTC1^TG^;Patz1^+/−^ mice show a thyroid cancer phenotype intermediate between that of RET/PTC1^TG^;Patz1^+/+^ and RET/PTC1^TG^;Patz1^−/−^ mice. 

A local lymph node metastasis was occasionally observed in a RET/PTC1^TG^;Patz1^+/−^ mouse carrying a solid variant of PTC. However, we did not systematically analyze lymph nodes or distant organs of all mice carrying thyroid tumors. Therefore, we cannot exclude that the number of metastases could be even higher than that one observed in our study. Future studies specifically focused on metastatization will be helpful to clarify this issue.

It is worth noting that the solid variant of PTC is associated with a less favorable prognosis than classical PTC [25]. Indeed, recent studies have shown that the presence of the solid component in PTC, regardless of the proportion, is associated with adverse clinical parameters and a shorter disease-free survival [26,27]. At a molecular level, the solid component is enriched in the expression of cancer stem cell markers ATP-binding cassette G2 (ABCG2) and multidrug resistance associated protein 1 (MRP1) that were absent or significantly lower expressed in the papillary component of the same tumor, whereas they are frequently overexpressed in ATC and are related to adverse clinical outcomes [28,29]. This is consistent with the idea that the solid component is less differentiated and may be a progression toward a poorly differentiated or anaplastic phenotype. 

The ability of PATZ1 to negatively regulate the EMT process [11,12] likely accounts for the higher malignant phenotype of the RET/PTC1^TG^ mice carrying a complete or a partial impairment of PATZ1 function. Consistently, it has been reported that EMT plays a key role in the development of ATC [30,31,32,33], and there are evidences in animal models that ATC can occur from preexisting PTC, passing through a PDTC [16,34]. Therefore, accordingly, we report that both E-cadherin and PATZ1 expression are absent in all PDTCs and ATCs developed by these mice, similar to that already described in human thyroid cancer, where PATZ1 expression is downregulated or lost in ATCs [11,12]. On these bases, we could speculate that PATZ1 loss is required to convert the classical variant of PTC into the solid one, accounting for the acquisition of a less differentiated and more aggressive phenotype. However, further experiments that more deeply investigate both upstream and downstream pathways involving PATZ1 in thyroid carcinogenesis will be necessary to fully address this hypothesis.

It is worth noting that Patz1-knockout mice, previously characterized in a mixed c57BL/6J × 129SvJ genetic background [22], did not spontaneously develop thyroid carcinomas during their lifespan [10], while they do with the new mixed FVB/N × c57BL/6J × 129SvJ genetic background. Indeed, we showed here that RET/PTC1^WT^;Patz1^+/−^ mice develop thyroid carcinomas even with a minor incidence and a longer latency than RET/PTC1^TG^;Patz1^+/−^ mice. This is consistent with previous reports in another mouse model of PTC, the transgenic mice expressing the *BRAF*^V600E^ oncogene under the control of the bovine thyroglobulin promoter, in which the genetic background of the mouse strain has a crucial role on phenotype determination [35,36]. A role of RET/PTC-RAS-BRAF signaling pathway as an initial event in thyroid carcinogenesis has been described [37]. The observed PATZ1 reduction during thyroid cancer development could be a consequence of an activation of the RET/PTC-RAS-BRAF signaling. Indeed, we have previously demonstrated that oncogenic RAS downregulates PATZ1 during thyroid carcinogenesis and that PATZ1 overexpression inhibits the malignant phenotype of thyroid cells transformed by the oncogenic Ras [24]. However, our data on Patz1^+/−^ mice that do not express RET/PTC1 in thyroid cells (Figure 1b) suggest that PATZ1 downregulation could be itself an initial event of thyroid carcinogenesis, independently from RET/PTC1.

In conclusion, even if with the limits of a small number of mice carrying the homozygous null mutation of Patz1 (due to the embryonic lethality) and the lack of a systematic study on the metastatic behavior, the results presented here provide compelling evidence that impairment of PATZ1 expression promote the occurrence and aggressiveness of thyroid tumors in RET/PTC1^TG^ mice, and per se may also be an initial event in thyroid carcinogenesis. Moreover, this genetically engineered mouse model, by recapitulating key features of the human ATC and aggressive PTC, may represent a helpful model for future research either in vivo or in vitro (by generating tumor-derived cell lines) in the development/evaluation of new therapeutic approaches.

## 4. Materials and Methods 

### 4.1. Animals

Heterozygous Patz1-knockout mice [22] of a mixed 129SvJ × c57BL/6J strain were crossed to FVB/N mice expressing RET/PTC1 under the control of the bovine thyroglobulin promoter [14]. The resulting RET/PTC1^TG^;Patz1^+/−^ and RET/PTC1^wt^;Patz1^+/−^ mice were bred to generate six genotypic groups: RET/PTC1^TG^;Patz1^+/+^, RET/PTC1^TG^;Patz1^+/−^, RET/PTC1^TG^;Patz1^−/−^, RET/PTC1^wt^;Patz1^+/+^, RET/PTC1^wt^;Patz1^+/−^, and RET/PTC1^wt^;Patz1^−/−^. For the comparison of histopathological analyses among RET/PTC1^TG^;Patz1^+/+^, RET/PTC1^TG^;Patz1^+/−^ and RET/PTC1^TG^;Patz1^−/−^, starting from 10 months up to 17 months, we sacrificed an equal number of mice (at least three) for each genotype at equal intervals of time and analyzed the thyroid phenotype. For tumor incidence curves, mice sacrificed before and after this time window (at 5, 18, 20–21, 23–24 months) were also included.

All mice were maintained under standardized nonbarrier conditions in the Laboratory Animal Facility of Istituto dei Tumori di Napoli (Naples, Italy). All studies were conducted in accordance with the 3Rs principle and Italian regulations for experimentations on animals (prot. no. 997/2014 approved by the Italian Ministry of Health on 3 March 2014). 

### 4.2. Genotyping

Three polymerase chain reactions (PCR) were performed on genomic DNA from tail clippings. Primers used to amplify a 203-bp DNA fragment from RET/PTC1 transgenic mice as previously reported (KD2: 5′-AGTTCTTCCGAGGGAATTCC-3′ and TPC4: 5′-GTCGGGGGGCATTGTCATCT-3′) [14]. A set of three primers was used to detect both normal and mutant *Patz1* alleles. To detect the Patz1-knockout allele, a 450-bp DNA fragment was amplified between a sequence inside the neomycin cassette and a sequence downstream of the replaced region of the *Patz1* gene, using the primers Pak5b: 5′-GCCTTCTTGACGAGTTCTTC-3′ and Pa3: 5′-CCACACCATCAAAGTTGG-3′. To detect the *Patz1* wild-type allele, a 385-bp DNA fragment was amplified between a sequence overlapping the replaced region in the knockout mutant and a sequence downstream of it and common to both wild-type and knockout alleles, using the primers PaN5: 5′-AAGCAAGTGGCTTGTGAG-3′ and Pa3. For all PCR cycling conditions were as follows: 1′ 95 °C followed by 35 cycles of 15″ 95 °C; 15″ 55 °C; 15″ 72 °C.

### 4.3. Histopathology and Immunohistochemistry

Histological evaluation of the thyroid gland was performed on all mice. Representative tissues were fixed overnight in 10% neutral buffered formalin, processed by routine methods, and embedded in paraffin. Sections (5 µm) were stained with ematoxylin and eosin. Blinded (i.e., without knowledge of genotype) histological evaluation included classification of tumor morphology (papillary, classical or solid, poor differentiated or ATC). Immunohistochemical staining was performed on 5 µm paraffin sections. Endogenous peroxidase was inhibited by 0.3% hydrogen peroxide in methanol for 30 min. For antigen retrieval, slides were microwaved in a DAKO autostainer in 0.01 M citric acid for 10 min and then quenched in 1% H_2_O_2_.

Polyclonal rabbit primary antibodies were against Ki-67 (ab 15580, 1:200, Abcam, Cambridge, UK), PATZ1 (custom ab R1P1, Primm [11]), and anti-E-cadherin (610181, BD Transduction Laboratories, BD Italia, Milan, Italy). The secondary antibody for all primary antibodies was biotinylated goat anti-rabbit antibody (Vector Laboratories, Burlingame, CA, USA). Specific binding was amplified using the streptavidin-biotin immunoperoxidase technique (DAKO). Chromogen reaction was developed with 3–3 diaminobenzidine (DAB) solution (DAKO), and nuclei were counterstained with Mayer’s hematoxylin. Negative controls were performed by omitting the primary antibody. Ki-67 expression was quantified by counting the percentage of immunopositive cells with respect to total cells, evaluating the mean of at least three fields for each tumor. At least four mice per group were included in this analysis. Conversely, immunohistochemical analysis of both PATZ1 and E-cadherin expression was only qualitative.

### 4.4. Statistical Analysis

Log-rank (Mantel–Cox) test was applied to analyze differences in Kaplan–Meier survival curves. Tumor distribution of the three *Patz1* genotypes of RET/PTC1 mice was analyzed to determine whether differences were statistically significant at 10 to 17 weeks of age. Pearson’s *x*^2^ test was performed on data expressed as percentage. Ordinary one-way ANOVA followed by Tukey’s multiple comparisons test was applied for Ki-67 analysis. All tests were assessed using GraphPad Prism 6 software, La Jolla (CA), USA. Statistical significance was indicated by *p* < 0.05. 

## Figures and Tables

**Table 1:** Histopathological diagnosis of tumors developed by RET/PTC1-Patz1 mice at 10–17 months of age.

Pathological Lesion	RET/PTC1^TG^;Patz1^+/+^ (*n* = 22)	RET/PTC1^TG^;Patz1^+/−^ (*n* = 24)	RET/PTC1^TG^;Patz1^−/−^ (*n* = 6)
Thyroid carcinoma	12 (54%)	14 (58%)	6 (100%)
Hyperplasia/goiter	7 (32%)	7 (29%)	0
Normal thyroid	3 (14%)	3 (12.5%)	0
